# The role of *IFITM3* in the growth and migration of human glioma cells

**DOI:** 10.1186/1471-2377-13-210

**Published:** 2013-12-27

**Authors:** Bing Zhao, Hongliang Wang, Gang Zong, Ping Li

**Affiliations:** 1 Department of Neurosurgery, the Second Affiliated Hospital of Anhui Medical University, 678 Fu Rong Road, Hefei 230601, China

**Keywords:** *IFITM3*, Glioma, RNAi, Growth, Migration

## Abstract

**Background:**

Interferon induced transmembrane protein 3 (*IFITM3*) is transcribed in most tissues and highly interferon-inducible. However, the role of *IFITM3* in cancer is still poorly understood.

**Methods:**

Expression levels ofIFITM3were analyzed in 60 glioma patients by immunohistochemistry (IHC). Following closely, we investigated the phenotype of *IFITM3* knockdown on glioma cell growth and tumorigenesis *in vitro* using lentivirus-mediated loss-of-function strategy.

**Results:**

Depletion of *IFITM3*in U251 cells dramatically inhibited cell proliferation and colony formation, which demonstrated that reduced IFITM3 protein levels could cause inhibition of tumorigenesis. Knockdown of *IFITM3* also induced cell cycle arrest in G0/G1 phase, especially in the sub-G1 phase representing apoptotic cells. In addition, the migration of U251 cells was visibly weakened after *IFITM3* knockdown, as determined by Transwell assay.

**Conclusions:**

Our findings provide new evidence that *IFITM3* plays an important role in glioma cell growth and migration, suggesting that silencing of *IFITM3* by RNA interference (RNAi) may be a potential approach to suppress glioma growth.

## Background

Glioma is the most common neurosurgical Nerve tumor [1,2]. At present, the prognosis of patients with malignant glioma remains very poor, and median survival is generally less than one year from the time of diagnosis, even in the most favorable situations, most patients die within two years [3,4]. Numerous studies have shown that gliomas develop as a result of genetic alterations that accumulate with tumor progression and therefore show a great morphological and genetic heterogeneity. Primary and secondary glioblastomas represent distinct entities evolving through different genetic pathways, molecular profile and response to therapy [5]. Therefore, new molecular targets and therapeutic strategies are urgently required for glioma therapy.

Interferon induced transmembrane protein 3 (*IFITM3*, also known as *1-8U*) was initially identified in a cDNA screen from INF-treated neuroblastoma cell back in 1984 [6] and cloned from a human lymphoid cell cDNA library [7]. *IFITM3* is transcribed in most tissues and is highly interferon-inducible [7,8]. Previous studies showed that *IFITM3* belongs to a family of murine genes [9], which are short, 2-transmembrane-domain proteins (5-18 kDa) with high core sequence similarity but divergent N- and C-termini. The human homologues (*IFITM1*, *IFITM2*, and *IFITM3*) are clustered on chromosome 11 within an 18-kb genomic sequence [7,10,11], and mediates cellular processes, including cell adhesion, immune-cell regulation, germ-cell homing and maturation, and bone mineralization [8,11-16].

Recent studies identified possible roles of *IFITM* genes in carcinogenesis. For example, *IFITM1* and *IFITM3* were shown to express at higher levels in astrocytoma cells than in normal astrocytes in mice [11,17,18]. Furthermore, *IFITM1* was identified as a key player in both the carcinogenesis and invasion in patients with glioma [19]. Also, *IFITM2* played a crucial role as a p53 independent pro-apoptotic gene in regulating cancer cellular pathways to death [20]. Researchers first isolated the *IFITM3* gene from tumor tissue and severely inflamed mucosa in the colons of patients with ulcerative colitis, describing it as a preferential marker for ulcerative colitis-associated colon cancer [21,22]. In addition, *IFITM3* expression has been found to be up-regulated in gastric cancer, colorectal tumors, and so on [23-25].

In this study, we showed the positive correlation between the expression levels of *IFITM3* and pathological grades of glioma by IHC. However, the precise function and underlying mechanism of *IFITM3* in glioma pathogenesis remain unclear. To study the role of *IFITM3* in glioma, we employed lentivirus-mediated short hairpin RNA (shRNA) to knock down *IFITM3* in human glioma cell line U251. The effects of *IFITM3* knockdown on cell growth and migration were investigated.

## Methods

### Materials

Dulbecco’s modified Eagle’s medium (DMEM) and fetal bovine serum (FBS) were obtained from Hyclone (Logan, Utah, USA). Lipofectamine 2000, TRIzol® Reagent was purchased from Invitrogen (Carlsbad, CA, USA). M-MLV Reverse Transcriptase was purchased from Promega (Madison, WI, USA; cat. M1705). All other chemicals were obtained from Sigma (St. Louis, MO, USA). The antibodies used were as follows: anti-IFITM3 (1:50 dilution; Sigma/SAB1410086).

### Immunohistochemistry (IHC)

We studied 60 glioma patients who had been surgically treated in Department of Neurosurgery, the Second Affiliated Hospital of Anhui Medical University, Hefei 230601, China. For IHC, 60 pairs of resected glioma tissues were fixed in 10% formalin solution and embedded in paraffin. Histological slices of 3 mm were prepared, then were deparaffined in xylene, and rehydrated with graded ethanol. Endogenous peroxidase was blocked with 0.3% H_2_O_2_ in methanol for 20 min at room temperature (RT). Following antigen retrieval, the sections were blocked with 5% BSA for 20 min at RT and then probed with 1:300 rabbit anti-IFITM3 at 4°C overnight. After washing, the sections were incubated with Histostain○R-Plus 3rd Gen IHC Detection Kit (Invitrogen/85–9073) at RT for 1 h, and visualized using the peroxidase conjugated streptavidin and diaminobenzidine, followed by counterstaining with Mayer’s haematoxylin. The IFITM3 antibody was replaced by PBS in negative controls. IHC staining were evaluated by a pathologist blinded to all clinical data. Samples were scored positive when more than 10% of the cells reacted with the anti-IFITM3 antibody and presented cytoplasm staining.

### Cell culture

Human glioma cell line U251 and human embryonic kidney cell line 293 T were obtained from American Type Culture Collection (ATCC). Cells were maintained in DMEM supplemented with 10% heat-inactivated FBS and 100 units/ml penicillin/streptomycin at 37°C in humidified atmosphere of 5% CO_2_.

### Construction of *IFITM3* shRNA lentivirus vector and cell infection

The following oligonucleotide was synthesized. The negative control small interfering RNA (siRNA) was 5′-TTCTCCGAACGTGTCACGT-3′. *IFITM3* siRNA was 5′-GCTGGAATTCATGAATCACACTGTCCAAAC-3′. The stem-loop-stem oligos (shRNAs) were synthesized, annealed, and ligated into the *Nhe* I/*Pac* I-linearized pFH-L vector. The lentiviral-based shRNA-expressing vectors were confirmed by DNA sequencing. The generated plasmids were named as pFH-L-sh*IFITM3* or -shCon. Recombinant lentiviral vectors and packaging vectors were then transfected into 293 T cells. Supernatants containing lentivirus expressing *IFITM3* shRNA or control shRNA were harvested 72 h after transfection. Then, the lentiviruses were purified using ultracentrifugation, and the titer of lentiviruses was determined. U251 cells were infected with the lentivirus constructs at multiplicity of infection (MOI) =10 and mock-infected cells were used as negative controls. To demonstrate specific knockdown of *IFITM3*, these experiments are also being repeated by using another two shRNAs (5′-CCAACTATGAGATGCTCAAGGCTCGAGCCTTGAGCATCTCATAGTTGGTTTTTT-3′ and 5′-CCTCATGACCATTCTGCTCATCTCGAGATGAGCAGAATGGTCATGAGGTTTTT-3′) against *IFITM3* to get comparable results.

### RT-PCR

Total RNA was extracted from U251 cells 5 days after infection using TRIzol® Reagent. cDNA was synthesized using M-MLV Reverse Transcriptase. In brief, a mixture containing 1.5 μg of total RNA, 0.75 μg oligo-dT primer (Shanghai Sangon) and nuclease-free water in a total volume of 13.5 μl was heated at 70°C for 5 min and then cooled on ice for another 5 min. The mixture was supplemented with 4 μl M-MLV buffer, 1.25 μl dNTP, 0.5 μl RNasin and 0.75 μl M-MLV-RT up to a final volume of 20 μl, followed by incubation at 42°C for 60 min.

### Real-time quantitative PCR

Real-time quantitative PCR analysis was performed using SYBR Green Master Mix Kit on BioRad connect Real-Time PCR platform. In brief, each PCR reaction mixture containing 10 μl of 2 × SYBR GreenMaster Mix, 1 μl of sense and antisense primers (5 μmol/μl) and 1 μl of cDNA (10 ng), was run for 45 cycles with denaturation at 95°C for 15 s, annealing at 60°C for 30 s and extension at 72°C for 30 s in a total volume of 20 μl. For relative quantification, 2^-ΔΔCT^ was calculated and used as an indication of the relative expression levels, which was calculated by subtracting CT values of the control gene from the CT values of *IFITM3*. The primer sequences for PCR amplification of *IFITM3* gene were 5′-TGTCCAAACCTTCTTCTCTCC-3′ and 5′-CGTCGCCAACCATCTTCC-3′. *β*-actin was applied as an internal control. The primer sequences of *β*-actin were 5′-GTGGACATCCGCAAAGAC-3′and 5′- AAAGGGTGTAACGCAA CTA-3′.

### MTT assay

U251 cells were trypsinized, resuspended, seeded into 96-well plate with a concentration of 2000 cells per well, and incubated at 37°C 3 days post lentivirus infection. The number of viable cells was measured at daily intervals (day 1, 2, 3, 4 and 5). At each time point, 10 μl of 5 mg/ml MTT (Dingguo Biotechnology) was added, and incubation was continued for 3 h. Then the medium was removed carefully and 100 μl of acidified isopropanol (in 0.01 M HCl) was added at the end of incubation. The absorbance was measured at 595 nm on the spectrophotometer.

### Colony formation assay

A total of 200 U251 cells were seeded in 6-well plates after 3 days of lentivirus infection. The medium was changed at regular time intervals. After 11 days of cultureat 37°C, the natural colonies were washed with PBS and fixed with 4% paraformaldehyde for 30 min at room temperature. The colonies were then stained with Giemsa for 10 min, washed with water and air-dried. The total number of colonies with more than 50 cells was counted under fluorescence microscopy.

### Fluorescence-activated cell sorting analysis

The cell cycle distribution was analyzed using flow cytometry with PI staining. In brief, 1.5 × 10^5^ cells that infected with lentivirus for 4 days were seeded in 6-cm dishes and allowed to culture for 40 h at 37°C. Cells were harvested after tripsinization, washed with PBS, and fixed with 70% cold ethanol. Cells were then collected by centrifugation, resuspended in PBS containing 100 μg/ml of DNase-free RNase and 40 μg/ml PI, and incubated for 1 h at 37°C. A total of 1.0 × 10^4^ fixed cells were analyzed by FACS can (Becton-Dickinson, Franklin Lakes, NJ, USA).

### Cell migration assay

U251 cells were infected with Lv-sh*IFITM3* for 4 days, and the *in vitro* migration ability was determined using a Transwell chamber (Corning, NY, USA). Briefly, cells were seeded into the upper chamber of the transwell plates (8.0 μm pore, Corning Costar, Cambridge, MA) with 3.0 × 10^4^ cells/well in 200 μl of serum-free medium, and 500 μl medium containing 10% FBS was added to the lower chamber as a chemoattractant. After incubation for 24 h at 37°C in 5% CO_2_, the cells remaining on the upper surface of the filter were removed, and those that invaded to the lower compartment were fixed with Methanol and stained with crystal violet. Cells were counted visually in 5 random fields under light microscope (10 × objective lens). In addition, invaded cells were dissociated, lysed and quantified at 570 nm using spectrophotometer.

### Statistical analysis

All data were expressed as mean ± SD of three independent experiments, in which each assay was performed in triplicate. The results were analyzed statistically using the chi-square test for the relationship between the incidence of immunoreactivity for *IFITM3* and the histological grades using GrafPad Prism version 5.0. The Student’s *t*-test was used to evaluate the differences between the control cells and *IFITM3* knockdown cells using SPSS 13.0 software. *P* < 0.05 was considered as statistically significant.

## Results and discussion

### Overexpression of IFITM3 in human glioma tissues

**Figure 1:**
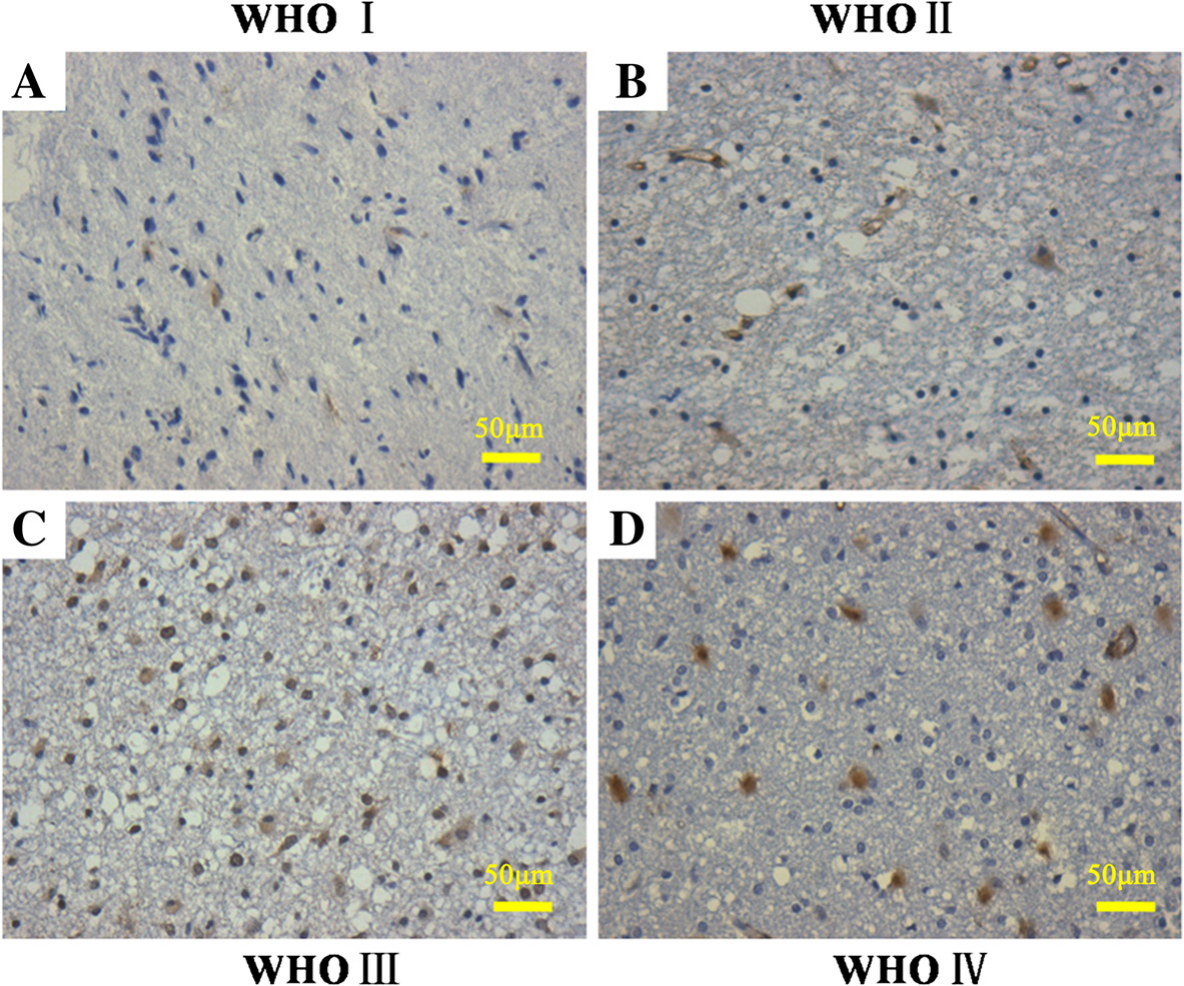
**Representative immunohistochemical staining for *IFITM3 *in patients with different grade glioma. A** pilocytic astrocytoma (WHO I); **B** astrocytoma (WHO II); **C** anaplastic astrocytoma (WHO III); **D** multiple glioblastoma (WHO IV). Original magnification 200 ×.

To evaluate the role of *IFITM3*in human glioma, IHC was performed to analyze the expression levels of IFITM3 in 60 glioma patients (grade I:7; grade II:25; grade III:17; grade IV:11). According to the World Health Organization (WHO) classification, low-grade gliomas encompass grade I and grade II tumors with the least malignant phenotypes, while high-grade gliomas encompass grade III (anaplastic astrocytoma, anaplastic oligoastrocytoma, and anaplastic oligodendroglioma) and grade IV (glioblastoma [GBM]) tumor with the most malignant phenotype [26]. Compared with the low grade glioma groups (Figure 1A and B), IFITM3 positive staining was mostly observed in high grade glioma (Figure 1C and D). IFITM3 expression level was increased with the histological grade of glioma (Table 1, P < 0.05). We also provided positive control and negative control in which the corresponding IFITM3 antibody was replaced by PBS, as shown in Additional file 1: Figure S1. These data clearly indicate that IFITM3 is specifically overexpressed in glioma tissues, and its high expression may contribute to the pathogenesis of glioma.

**Table 1:** **The relationship between the incidence of immunoreactivity for *IFITM3 *** **in human glioma tissue specimens and the histological grades**

**Sample**	**N**	**Expression of IFITM3**		**Chi-square**	**P value**
		**Negative**	**Positive**		
Low-grade gliomas (I, II)	28	7 (25.0%)	21 (75.0%)	4.118	0.0424
High-grade gliomas (III, IV)	32	2 (6.3%)	30 (93.8%)		

N: number of samples.

### Knockdown of *IFITM3* in U251 cells by lentivirus infection

**Figure 2:**
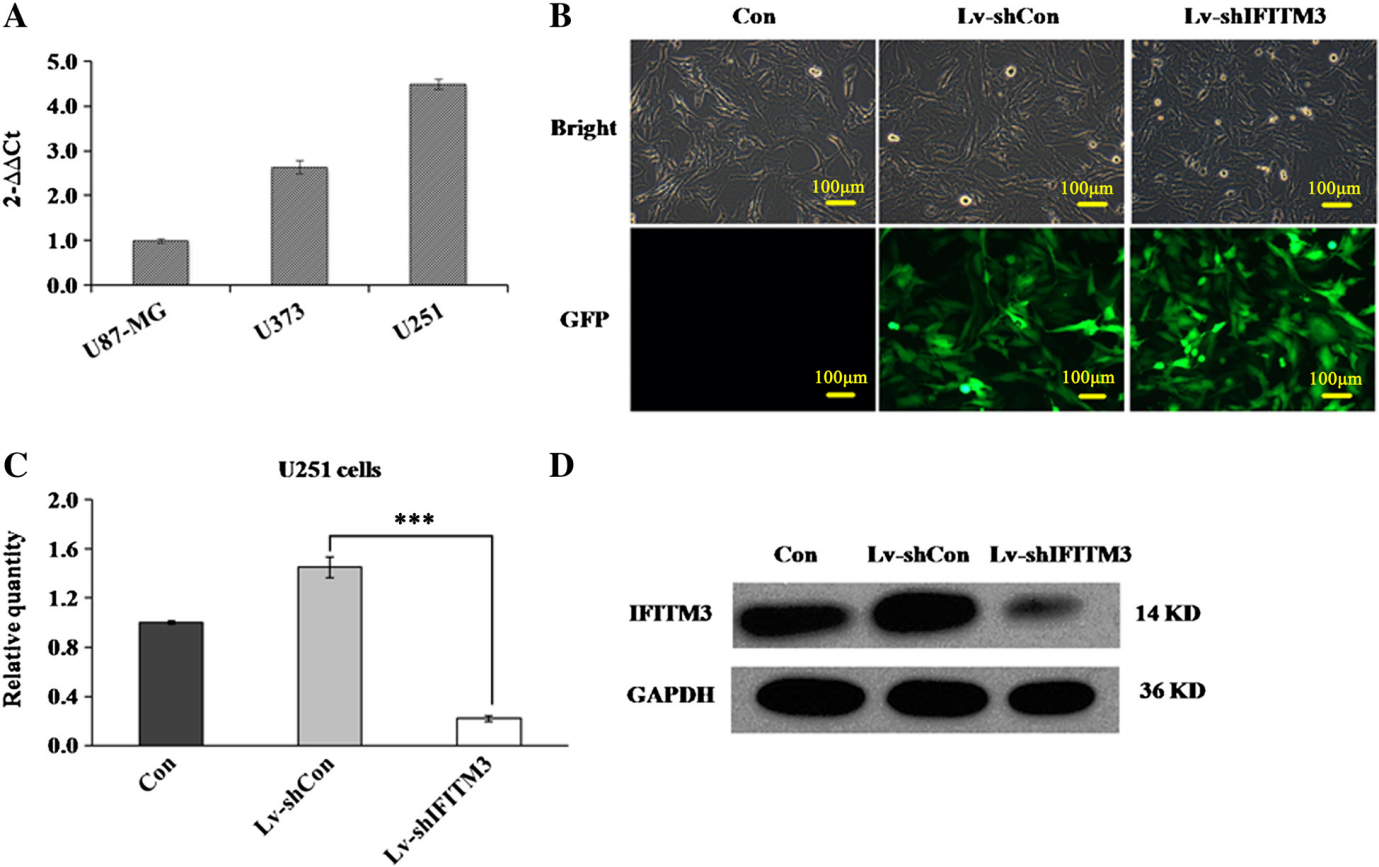
**Lentivirus-mediated gene silencing of *IFITM3 *in glioma cells. (A)** Quantitative RT-PCR analysis for *IFITM3* mRNA levels in U251, U87-MG and U373 glioma cells; **(B)** Determination of infection efficiency in the human glioma cells. Representive images of U251 cells after 3 days of lentivirus infection were shown (10× objective lens). **(C)** Expression analysis of *IFITM3* mRNA by qRT-PCR in uninfected U251 cell (Con), cells infected with Lv-shCon, and cells infected with Lv-sh*IFITM3*. The *β-actin* gene is the internal controls for qRT-PCR. Significant difference from Lv-shCon (*P* < 0.001). **(D)** Expression analysis of IFITM3 protein in uninfected group, Lv-shCon group and Lv-shIFITM3 group by western blot.

Human glioma cells of different transformation degrees, as represented by U373 astrocytoma (WHO grade III), U87-MG and U251 glioblastoma multiforme (WHO grade IV) were selected to detect *IFITM3* expression. As shown in Figure 2A, high expression levels of *IFITM3* were observed in all three cells. U251 cells, derived from a high-grade glioblastoma, were used for the following loss-of-function investigation. We constructed a lentiviral vector system to express siRNA targeting *IFITM3* and to express GFP as a reporter gene. To determine the efficacy of recombinant lentiviruses, U251 cells infected with Lv-sh*IFITM3* and Lv-shCon were observed under a fluorescence microscope. More than 90% of the cells expressed GFP after 72 h infection (Figure 2B), which indicated high-efficiency infection by Lv-sh*IFITM3*. To verify that the *IFITM3* gene was silenced by Lv-sh*IFITM3*, we determined the mRNA levels in uninfected, Lv-shCon and Lv-sh*IFITM3*-infected cells by qRT-PCR. Cell infected with Lv-sh*IFITM3* exhibited significant decreased *IFITM3* mRNA levels, by 84.8% reduction in U251 cells, compared with Lv-shCon-infected and uninfected cells (Figure 2C, *P* < 0.001). The similar result was observed in U251 cells infected with another two shRNA stargeting *IFITM3*, respectively (Additional file 2: Figure S2 A, *P* < 0.001). To confirm the silencing of IFITM3, western blot was used using IFITM3 antibodies. Compared with uninfected and Lv-shCon infected cells, the IFITM3 protein level was significantly decreased in U251 cells infected with the Lv-sh*IFITM3* (Figure 2D). Similarly, only weak band was detected in U251 cells infected with another two shRNAs targeting *IFITM3*, respectively, while the high expression of IFITM3 was not affected in cells infected with Lv-shCon compared with control cells (Additional file 2: Figure S2 B).

### Knockdown of *IFITM3* inhibited cell proliferation

**Figure 3:**
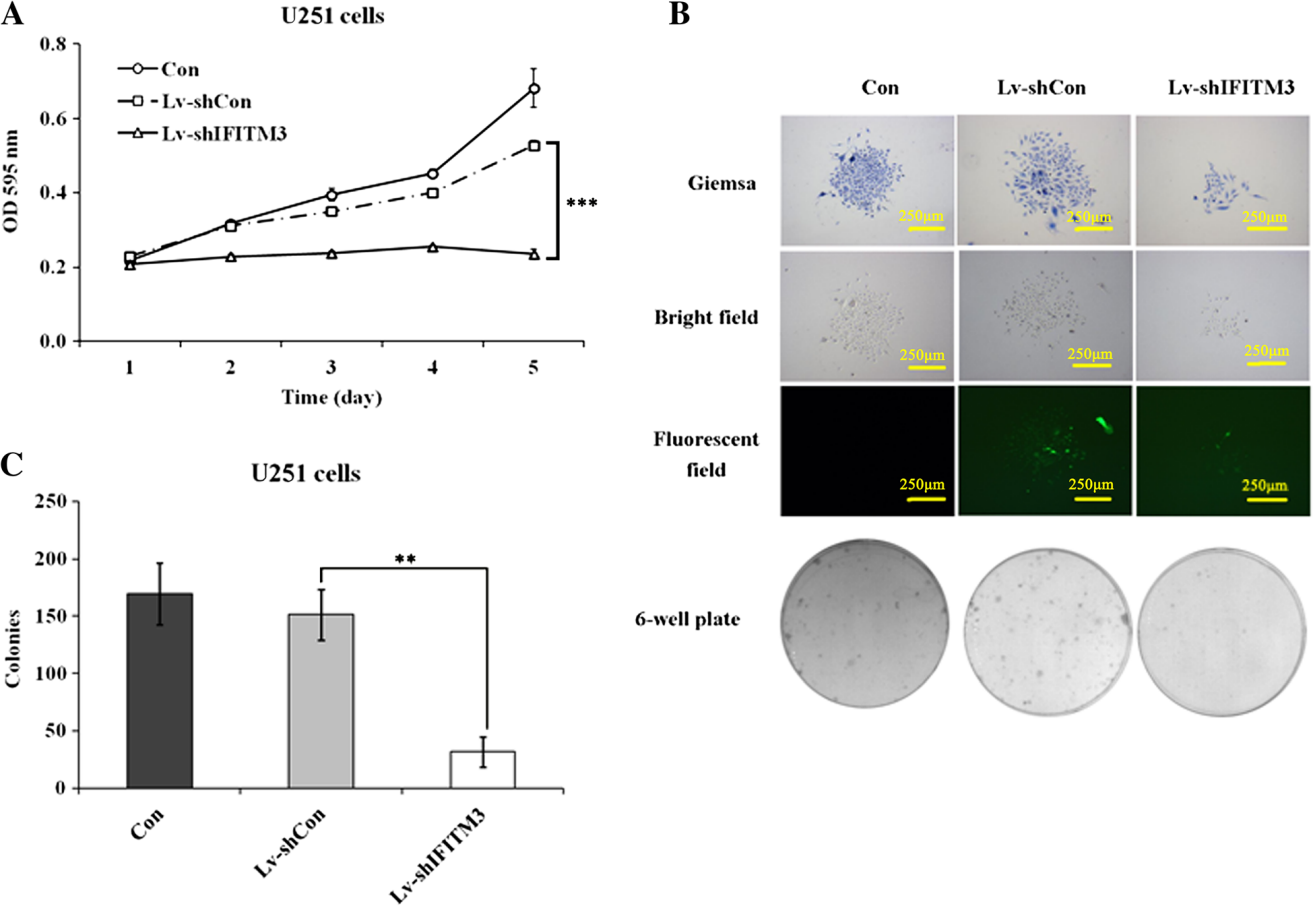
**Lv-sh*IFITM3 *infection inhibits the proliferation and colony formation of U251 cells. (A)** In vitro proliferation assay of U251 cells without infection (Con), cells infected with Lv-shCon, and cells infected with Lv-sh*IFITM3*. Cell proliferation in the Lv-sh*IFITM3* group was significantly inhibited, as demonstrated by MTT assay. Significant difference from Lv-shCon (*P* < 0.001). **(B)** Images recorded under light microscope and fluorescence microscope, representing the size and number of colonies in each group of cells. **(C)** Statistical analysis of the number of colonies with Giemsa staining. Significant difference from Lv-shCon (*P* < 0.01).

MTT assay was performed to investigate the effect of *IFITM3* knockdown on cell proliferation. The growth curve obtained from the MTT assay indicated that the proliferative ability of Lv-sh*IFITM3*-infected cells was significantly decreased, by 55.2% reduction in U251 cells, when compared with that of Lv-shCon-infected cells (*P* < 0.001, Figure 3A). The similar results could be seen in Additional file 2: Figure S2 C, the proliferative ability of Lv-shIFITM3-S2-infected and Lv-shIFITM3-S3-infected cells was significantly decreased, respectively. These results indicate that knockdown of *IFITM3* by RNAi could inhibit the proliferation of glioma cells.

### Downregulation of *IFITM3* suppressed colony formation

To determine whether *IFITM3* has any impact on the colony-forming capacity of glioma cells, colony formation assay was performed. Our data indicated that the number and size of colonies formed fromLv-sh*IFITM3*-infected cells were strongly decreased, by 78.8% reduction in U251 cells, compared with Lv-shCon-infected cells (Figure 3B and C, *P* < 0.01), suggesting that the reduced expression of *IFITM3* could significantly inhibit colony formation in glioma cells.

### Depletion of *IFITM3* induced cell cycle arrest as well as apoptosis

**Figure 4:**
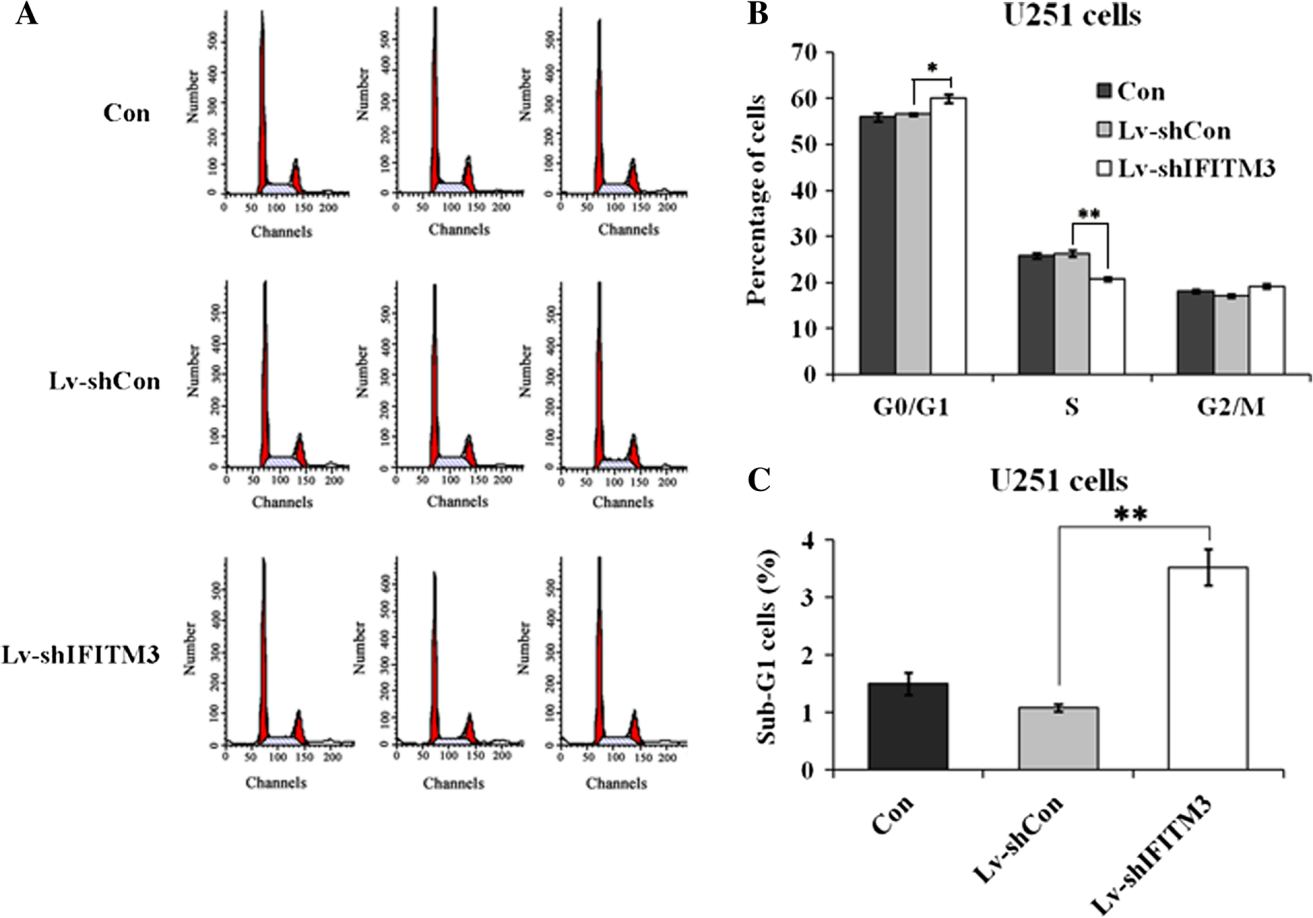
**Knockdown of *IFITM3 *blocks cell cycle progression in U251 cells. (A)** Cell cycle distribution of U251 cells was analyzed by flow cytometry. **(B)** Compared with Lv-shCon, the population of cells in the G0/G1 and S phase were increased the Lv-sh*IFITM3* group (*P* < 0.05, *P* < 0.01). **(C)** Percentage of apoptotic cells showed a significant increase in the Lv-sh*IFITM3* group. Significant difference from Lv-shCon (*P* < 0.01).

To elucidate whether knockdown of *IFITM3* inhibits cell growth by affecting cell cycle progression, we assessed the cell cycle distribution in U251 cells by flow cytometry (Figure 4A). As shown in Figure 4B, compared to control groups, cell population in the Lv-sh*IFITM3* group displayed a significant decrease in S phase (Lv-shCon 56.58% ± 0.36% *vs* 60.07% ± 0.90%, *P* < 0.01), and a significant increase in G0/G1 phase (Lv-shCon 56.58% ± 0.36%*vs* 60.07% ± 0.90%, *P* < 0.05). Accumulation of cells in the sub-G1 fraction was clearly observed in the Lv-sh*IFITM3* group compared to Lv-shCon group (Lv-shCon 1.09% ± 0.07%*vs* 3.35% ± 0.32%, *P* < 0.01) (Figure 4C). Taken together, these data suggest that *IFITM3* could modulate cell growth via cell cycle regulation as well as apoptosis.

### Downregulation of *IFITM3* attenuated cell migration

**Figure 5:**
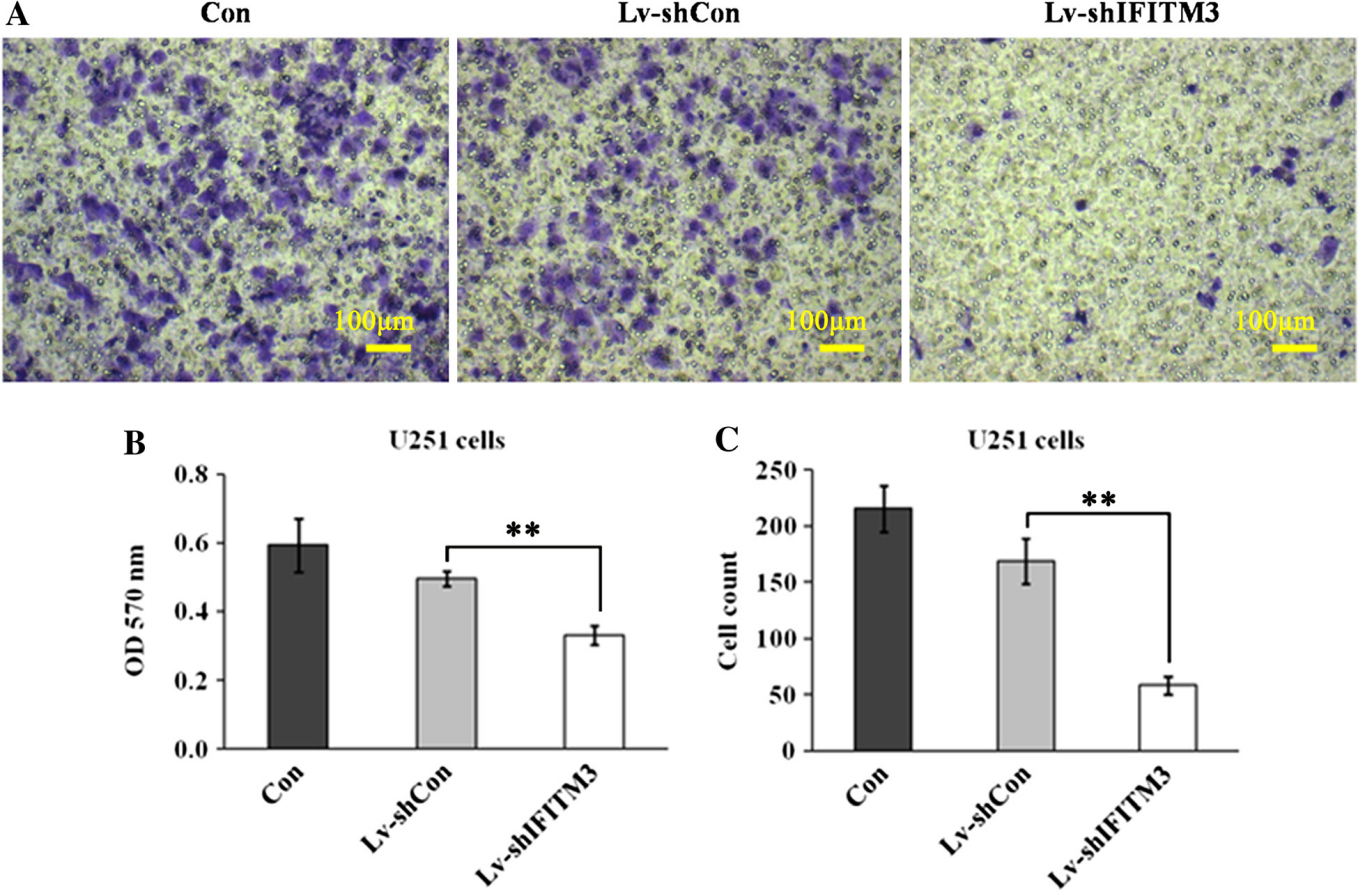
**Lv-sh*IFITM3 *infection induces reduction in migration capacity of U251 cell. (A)** Images recorded under light microscope, representing that *IFITM3* knockdown could reduce the motility of U251 cells. **(B and C)** Statistical analysis of OD570 and cell count in U251 cells after Lv-sh*IFITM3* infection for 4 days. Significant difference from Lv-shCon (*P* < 0.01).

To detect whether knockdown of *IFITM3* affects the migration of glioma cells, Transwell migration assay was performed to assess the proportion of U251 cells which migrated through polycarbonate membranes following 4 days of lentivirus infection. We found that *IFITM3* knockdown reduced the number of invaded cells accounting for 65.5% reduction in U251 cells (Figure 5A and B, *P* < 0.01). In addition, the colorimetric assay showed that the motility capability of U251 cells were retained after Lv-sh*IFITM3* infection (*P* < 0.01, Figure 5C).

## Conclusions

Glioma is triggered by a series of point mutations and genetic alterations that progressively cause normal cells to transform into precancerous cell which could become more dysplastic, resulting in carcinoma foci [27]. At present, the identification of useful markers for the diagnosis of human glioma is a major goal in cancer research, which also provides valuable information in tumor pathogenesis. We analyzed the expression of IFITM3 in human glioma specimens by immunohistochemistry and found the expression levels of IFITM3 were up-regulated in varying degrees and positively correlated with glioma of pathological grade I ~ II and III ~ IV (P < 0.05). Meanwhile, recent studies have also shown that *IFITM3* plays a prominent role in tumor development and can be used as a tumor biomarker [21-25]. However, the precise role of *IFITM3* in glioma pathogenesis remains unknown.

In recent few years the discovery of RNAi, a powerful tool to induce loss-of-function phenotypes through the posttranscriptional silencing of gene expression, has provided new possibilities for cancer therapy [28-30]. In our study, lentivirus-mediated RNAi was used to knock down *IFITM3* in human glioma cells. The siRNA targeting *IFITM3*, which expressed from the recombinant lentivirus, induced efficient and specific inhibition of endogenous *IFITM3* mRNA in U251 cells. Simultaneously, depletion of *IFITM3* led to significant reduced cell proliferation, colony formation and cell migration in U251 cells. Thus, this study confirms a crucial role of *IFITM3* in glioma tumorigenesis, suggesting *IFITM3* as an oncogene in human glioma.

Moreover, cell cycle analysis indicated that knockdown of *IFITM3* remarkably induced U251 cells accumulation in G0/G1 phase, especially in sub-G1 phase, which represents cell apoptosis [31]. Together, *IFITM3* may promote human glioma growth by inducing cell cycle arrest and apoptosis. Based on the *IFITM3* belong to 2-transmembrane-domain proteins (5-18 kDa) with high core sequence similarity but divergent N- and C-termini, we suggest *IFITM3* as a growth regulator in glioma, and it may be involved in the control of transport essential raw materials for DNA and enzymic synthesis process in cell cycle progression.

However, some studies indicated *IFITM3* plays an important role in inhibiting tumor development. For instance, El-Tanani et al. [32] found that *IFITM3* in breast cancer physically interacts with *OPN* and reduces *OPN* mRNA expression, which mediates cancer cell adhesion, cell invasion and colony formation. Similar situation has come under observation in human melanoma, and it is deemed dynamic promoter methylation adds an additional layer of complexity to the IFN-α key response genes like *IFITM3* be required for comprehensive control of the IFN-α response [33,34]. Therefore, we think the roles of *IFITM3* in various cancers possibly depend on tumor growth microenvironment. Under normal physiological circumstances, *IFITM3* could be in a balance regulating by different signal pathways.

Furthermore, local invasion is one of the primary reasons for clinical treatment failure in malignant gliomas. Recently, there has been increasing evidence regarding specific molecules that determine the aggressiveness and invasion potential of high grade astrocytic tumors [35]. In our study, we found *IFITM3* knockdown significantly reduced the migration capacity of U251 cells, indicating that *IFITM3* might play an essential role in glioma metastasis. However, further experiments are needed to elucidate the mechanism of *IFITM3* in glioma cell growth and migration.

To sum up, knockdown of *IFITM3* by RNAi successfully reduced cell proliferation and migration, and promoted apoptosis in glioma cells. Our results provide new evidence for the involvement of *IFITM3* in carcinogenesis, and suggest that RNAi-directed *IFITM3* silencing may be a potent therapeutic tool in glioma.

## Consent

Written informed consent was obtained from the patient for the publication of this report and any accompanying images.

## Competing interests

The authors declare that they have no competing interests.

## Authors’ contributions

BZ conceived, coordinated and designed the study, and contributed to the acquisition, analysis and interpretation of data and drafted the manuscript. HW and GZ performed the experiment and involved in drafting the article. BZ and PL accepts full responsibility for the work and/or the conduct of the study, had access to the data, and oversaw the decision to publish. All authors read and approved the final manuscript.

## Pre-publication history

The pre-publication history for this paper can be accessed here:

http://www.biomedcentral.com/1471-2377/13/210/prepub

## Supplementary Material

Additional file 1: Figure S1 Positive control and negative control for the specificity of the anti-IFITM3 antibody. Representative immunohistochemical staining for IFITM3 in glioma (A) and colon cancer tissues (C). Immunohistochemical staining for the corresponding IFITM3 antibody was replaced by PBS in glioma tissues (B). Click here for file

Additional file 2: Figure S2 (A) Determination of knockdown efficiency in the U251 cells infected with Lv-shIFITM3-S2 and Lv-shIFITM3-S3 by qRT-PCR. Significant difference from Lv-shCon (*P* < 0.001). (B) Expression analysis of IFITM3 protein in uninfected group, Lv-shCon group and Lv-shIFITM3-S2 group and Lv-shIFITM3-S3 group by western blot. (C) The effect of IFITM3-S2 and IFITM3-S3 on the proliferation of U251 cells. Significant difference from Lv-shCon (*P* < 0.001). Click here for file
