# Supplementary figures and images for: The role of IFITM3 in the growth and migration of human glioma cells
Source: BMC Neurol. 2013 Dec 27;13:210. doi: 10.1186/1471-2377-13-210 (PMC3883121; doi:10.1186/1471-2377-13-210)

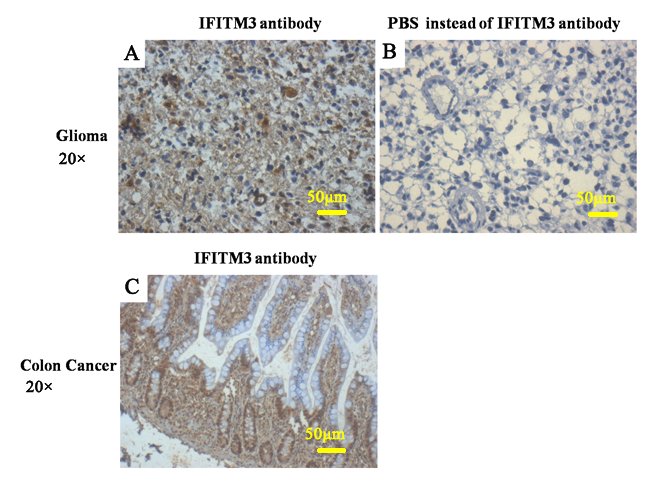

Supplement: Additional file 1: Figure S1 — Positive control and negative control for the specificity of the anti-IFITM3 antibody. Representative immunohistochemical staining for IFITM3 in glioma (A) and colon cancer tissues (C). Immunohistochemical staining for the corresponding IFITM3 antibody was replaced by PBS in glioma tissues (B). [file 1471-2377-13-210-S1.tiff]

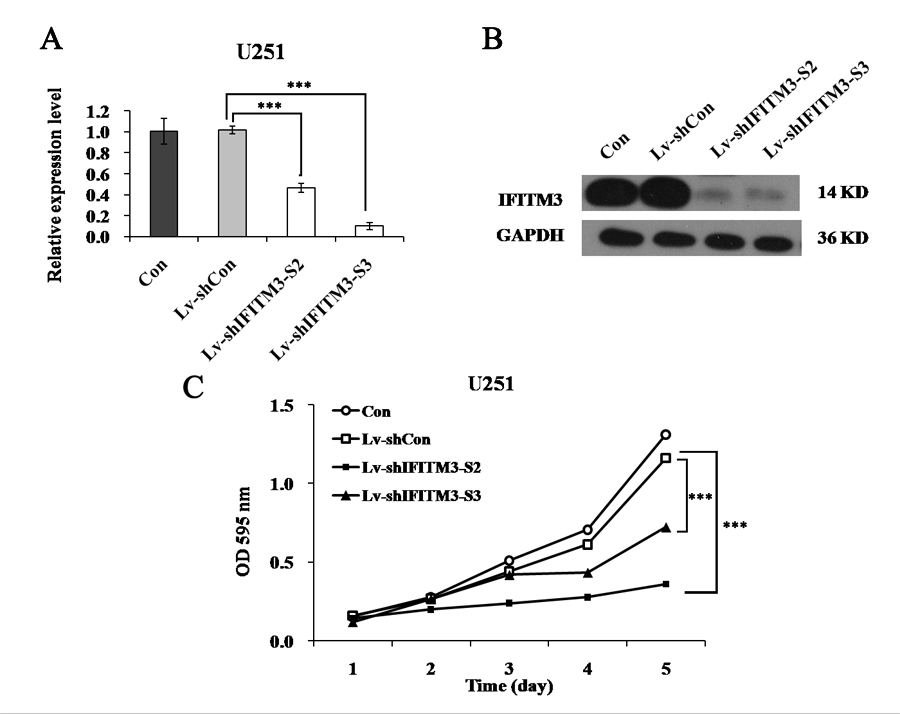

Supplement: Additional file 2: Figure S2 — (A) Determination of knockdown efficiency in the U251 cells infected with Lv-shIFITM3-S2 and Lv-shIFITM3-S3 by qRT-PCR. Significant difference from Lv-shCon (P < 0.001). (B) Expression analysis of IFITM3 protein in uninfected group, Lv-shCon group and Lv-shIFITM3-S2 group and Lv-shIFITM3-S3 group by western blot. (C) The effect of IFITM3-S2 and IFITM3-S3 on the proliferation of U251 cells. Significant difference from Lv-shCon (P < 0.001). [file 1471-2377-13-210-S2.tiff]
